# The global burden of disease study 2013: What does it mean for the NTDs?

**DOI:** 10.1371/journal.pntd.0005424

**Published:** 2017-08-03

**Authors:** Jennifer R. Herricks, Peter J. Hotez, Valentine Wanga, Luc E. Coffeng, Juanita A. Haagsma, María-Gloria Basáñez, Geoffrey Buckle, Christine M. Budke, Hélène Carabin, Eric M. Fèvre, Thomas Fürst, Yara A. Halasa, Charles H. King, Michele E. Murdoch, Kapa D. Ramaiah, Donald S. Shepard, Wilma A. Stolk, Eduardo A. Undurraga, Jeffrey D. Stanaway, Mohsen Naghavi, Christopher J. L. Murray

**Affiliations:** 1 Department of Pediatrics and Molecular Virology and Microbiology, National School of Tropical Medicine, Baylor College of Medicine, Houston, Texas, United States of America; 2 James A. Baker III Institute for Public Policy, Rice University, Houston, Texas, United States of America; 3 Texas Children’s Hospital Center for Vaccine Development, Houston, Texas, United States of America; 4 Scowcroft Institute of International Affairs, Bush School of Government and Public Service, Texas A&M University, College Station, Texas, United States of America; 5 Department of Biology, Baylor University, Waco, Texas, United States of America; 6 Institute for Health Metrics and Evaluation, University of Washington, Seattle, Washington, United States of America; 7 Department of Public Health, Erasmus MC, University Medical Center Rotterdam, Rotterdam, The Netherlands; 8 London Centre for Neglected Tropical Disease Research, Department of Infectious Disease Epidemiology, Imperial College London, London, United Kingdom; 9 University of California, San Francisco, San Francisco, California, United States of America; 10 Department of Veterinary Integrative Biosciences, College of Veterinary Medicine and Biomedical Sciences, Texas A&M University, College Station, Texas, United States of America; 11 Department of Biostatistics and Epidemiology, College of Public Health, University of Oklahoma Health Sciences Center, Oklahoma City, Oklahoma, United States of America; 12 International Livestock Research Institute, Nairobi, Kenya; 13 Institute of Infection and Global Health, University of Liverpool, Liverpool, United Kingdom; 14 School of Public Health, Imperial College London, London, United Kingdom; 15 Department of Epidemiology and Public Health, Swiss Tropical and Public Health Institute, Basel, Switzerland; 16 University of Basel, Basel, Switzerland; 17 Schneider Institutes for Health Policy, Brandeis University, Waltham, Massachusetts, United States of America; 18 Center for Global Health and Diseases, Case Western Reserve University School of Medicine, Cleveland, Ohio, United States of America; 19 Department of Dermatology, Watford General Hospital, Watford, Herts, United Kingdom; 20 Consultant on Lymphatic Filariasis, Tagore Nagar, Pondicherry, India; National Institute of Parasitic Diseases China CDC, CHINA

*The new Global Burden of Disease Study 2013 has identified some key trends in the major neglected tropical diseases, many with public health and policy implications*.

The Global Burden of Disease Study (GBD) is a landmark initiative that systematically quantifies the prevalence, morbidity, and mortality for hundreds of diseases, injuries, and risk factors of global health importance. For the neglected tropical diseases (NTDs), the GBD 2010 confirmed a high disease burden for the 17 major NTDs prioritized by the World Health Organization (WHO) as well as for selected conditions also recognized as NTDs by *PLOS Neglected Tropical Diseases*, including amoebiasis, cholera, cryptosporidiosis, typhoid and paratyphoid fevers, trichomoniasis, venomous animal contact, and scabies (referred to here as “additional NTDs”) [1]. The GBD 2013 is intended to be the first in a series of annual updates for the GBD studies, with its initial results published in 2015 in *The Lancet* [2–4]. Here, we review information on the NTDs published in the GBD 2013 capstone papers [2–4] and present new NTD data and updated burden estimates from the GBD 2013 study and new country-specific estimates. We show key outputs of GBD 2013 including country-specific estimates of prevalence or incidence and health-gap metrics for the aforementioned NTDs.

## Global prevalence and trends for NTDs in 2013

GBD 2013 estimates suggest that NTDs are among the world’s most common conditions, with more than 2 billion prevalent NTD infections globally in 2013 (Table 1). The 3 major intestinal helminth infections—ascariasis, trichuriasis, and hookworm infection—account for 1.75 billion of those cases, comprising more than three-quarters of the total prevalent NTD infections. Also highly prevalent are schistosomiasis, foodborne trematodiases, lymphatic filariasis (LF), and onchocerciasis as well as dengue fever [3]. In total, there are 2.3 billion cases of the WHO-prioritized NTDs plus “other NTDs” globally in 2013 and at least 160 million cases of additional neglected diseases.

**Table 1:** Prevalent cases of NTDs in 2013 and percent change from 1990 to 2013 according to the Global Burden of Disease Study (GBD) 2013 [3].

**Disease**	**Prevalent cases (in millions) in 2013**	**Percent change since 1990**
Ascariasis	804.4	−25.5%
Trichuriasis	477.4	−11.6%
Hookworm	471.8	−5.1%
Schistosomiasis	290.6	30.9%
Foodborne trematodiases	80.2	51.1%
Dengue*^†^	58.4	610.9%
Lymphatic filariasis	43.9	−32.1%
Onchocerciasis	17.0	−31.2%
Chagas disease	9.4	22.4%
Cutaneous/mucocutaneous leishamaniasis	3.9	174.2%
Trachoma^†^	2.4	−39.2%
Cysticercosis^†^	1.0	−26.3%
Cystic echinococcosis^†^	0.8	−15.4%
Leprosy	0.7	61.3%
Visceral leishmaniasis	0.1	35.1%
Rabies*^†^	0.02	−40.4%
African trypanosomiasis	0.02	−71.1%
Other NTDs	59.7	−5.0%
**Total cases**	**2,322**	**NA**
**Additional NTDs**	**Prevalent cases (in millions) in 2013**	**Percent change since 1990**
Trichomoniasis	67.1	45.6%
Scabies	66.1	24.8%
Typhoid fever*	11.0	−19.9%
Paratyphoid fever*	6.4	−27.9%
Venomous animal contact*	5.5	−2.7%
Cholera*	2.3	6.1%
Cryptosporidiosis*	1.4	−19.4%
Amoebiasis*	0.4	17.0%
**Total cases of additional neglected diseases**	**160.2**	**NA**

* Incident cases in 2013 rather than prevalent cases.

† Symptomatic cases only.

NOTE: For information on percent change calculations, see GBD 2013 capstone paper on incidence, prevalence, and years lived with disability (YLDs) [3]. All data presented in this table (except for rabies, cholera, cryptosporidiosis, and amoebiasis) are also available from the Institute for Health Metrics and Evaluation (IHME) website and were previously published in [3]. **Abbreviations:** NA, non-applicable

**Fig 1:**
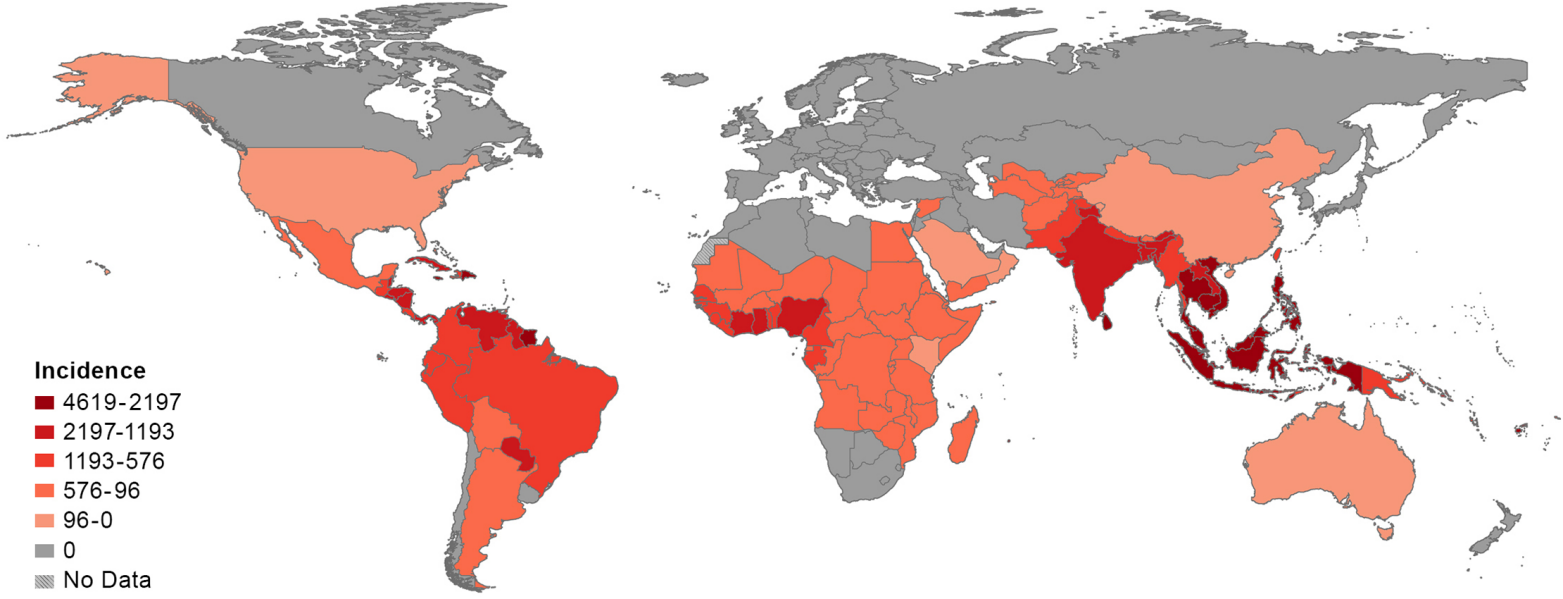
Global dengue incidence per 100,000 person-years in 2013. **Adapted from Stanaway et al. [40].** NOTE: No estimates are available for Western Sahara as it was not a modeled location in the Global Burden of Disease Study (GBD) 2013.

**Fig 2:**
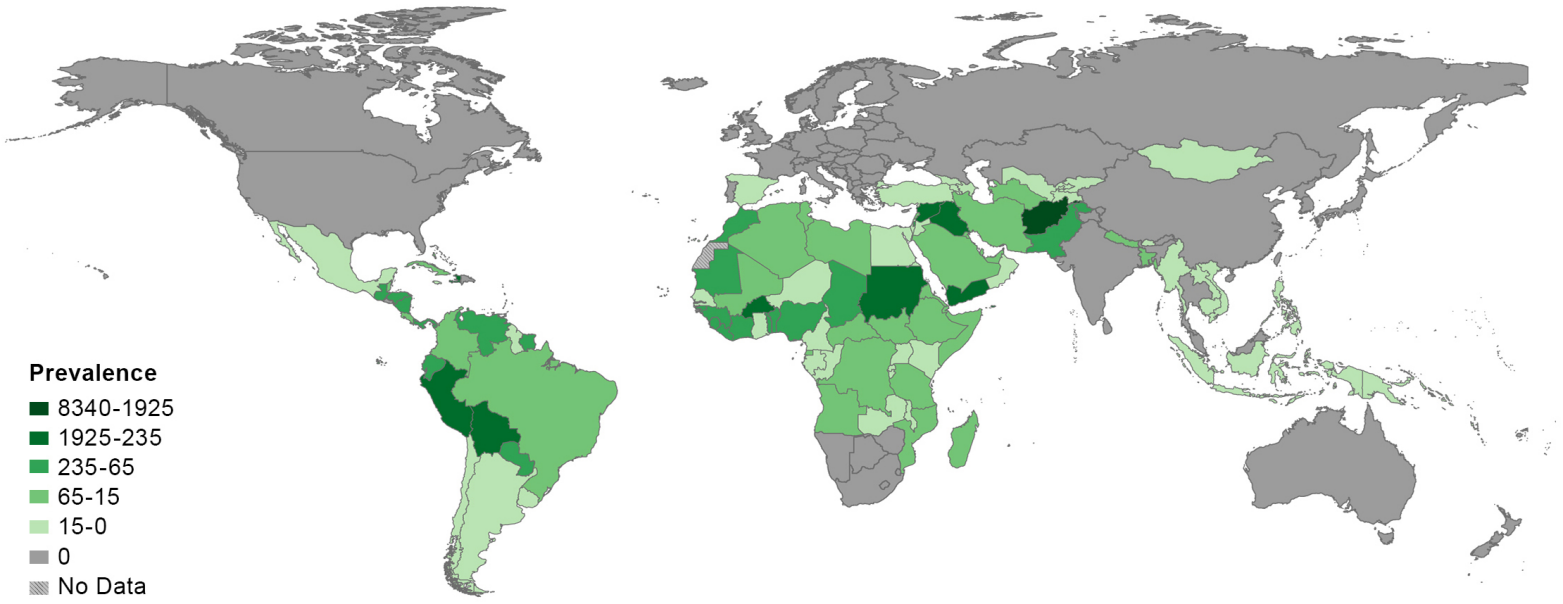
Global cutaneous/mucocutaneous leishmaniasis prevalence per 100,000 population in 2013. NOTE: No estimates are available for Western Sahara as it was not a modeled location in the Global Burden of Disease Study (GBD) 2013.

GBD 2013 reveals some major and notable changes in prevalence or incidence of these diseases since 1990. The most notable is a 610% increase in dengue fever incidence, consistent with the widespread emergence of this disease in Asia, Africa, and the Americas beyond what would be expected due to changes in population demographics. Overall, the major Southeast Asian countries exhibit the highest incidence, as do selected countries in the Caribbean, Central America, and tropical areas of South America (Fig 1). South Asia and West African countries bordering the Gulf of Guinea also exhibit high incidence. In addition, there has been a nearly 175% increase in the estimated number of prevalent cases of cutaneous and mucocutaneous leishmaniasis, which is associated with the major increases from 1990–2013 in the conflict areas of the Middle East and Central Asia (Afghanistan: 138%, Iraq: 1,293%, and Syria: 1,660%) and in East Africa (Sudan: 2,009%). The marked increases in these countries may be linked to conflict-associated collapsed health systems and/or increases in reporting rates over time [5–8]. The increase in prevalence of cutaneous and mucocutaneous leishmaniasis by country is shown in Fig 2. Marked increases of over 50% in the estimated number of prevalent cases were also noted for leprosy and foodborne trematodiases.

GBD 2013 found that there have been substantial reductions (approximately 30%–40%) in prevalent cases of trachoma-attributable vision impairment, LF, and onchocerciasis. There were also considerable reductions in ascariasis, which a previous analysis has associated with trends in China [9]. These changes relate to increases in mass drug administration (MDA) programs over the last decade [10] both as school-based and community-based programs, the latter also due to the scaling up of LF and onchocerciasis control and elimination efforts (ascaris is susceptible both to benzimidazoles and to ivermectin). Considering the progress of these control and elimination programs, we expect to see a further reduction in disease burden and possibly elimination of disease transmission in the coming years in many countries [11–14]. However, to date, there has been no substantial impact on the prevalence of schistosomiasis and only modest impact for 2 of the soil-transmitted helminth infections (STHs)—hookworm and trichuriasis.

Finally, other major trends noted in GBD 2013 include a 71% reduction in the number of cases of human African trypanosomiasis (HAT) infection. HAT is another NTD for which elimination may be plausible—especially the Gambian form—through case detection and treatment [15]. There have also been reductions in the number of cases of disease from rabies, cysticercosis, and cystic echinococcosis since 1990. However, cysticercosis and cystic echinococcosis still cause substantial morbidity (years lived with disability [YLDs]) and rabies continues to cause substantial mortality (see section Death and DALY Trends for NTDs in 2013 below for more information in YLDs, years of life lost [YLLs], disability-adjusted life years [DALYS], and deaths).

## Regional prevalence and incidence distribution in 2013

**Fig 3:**
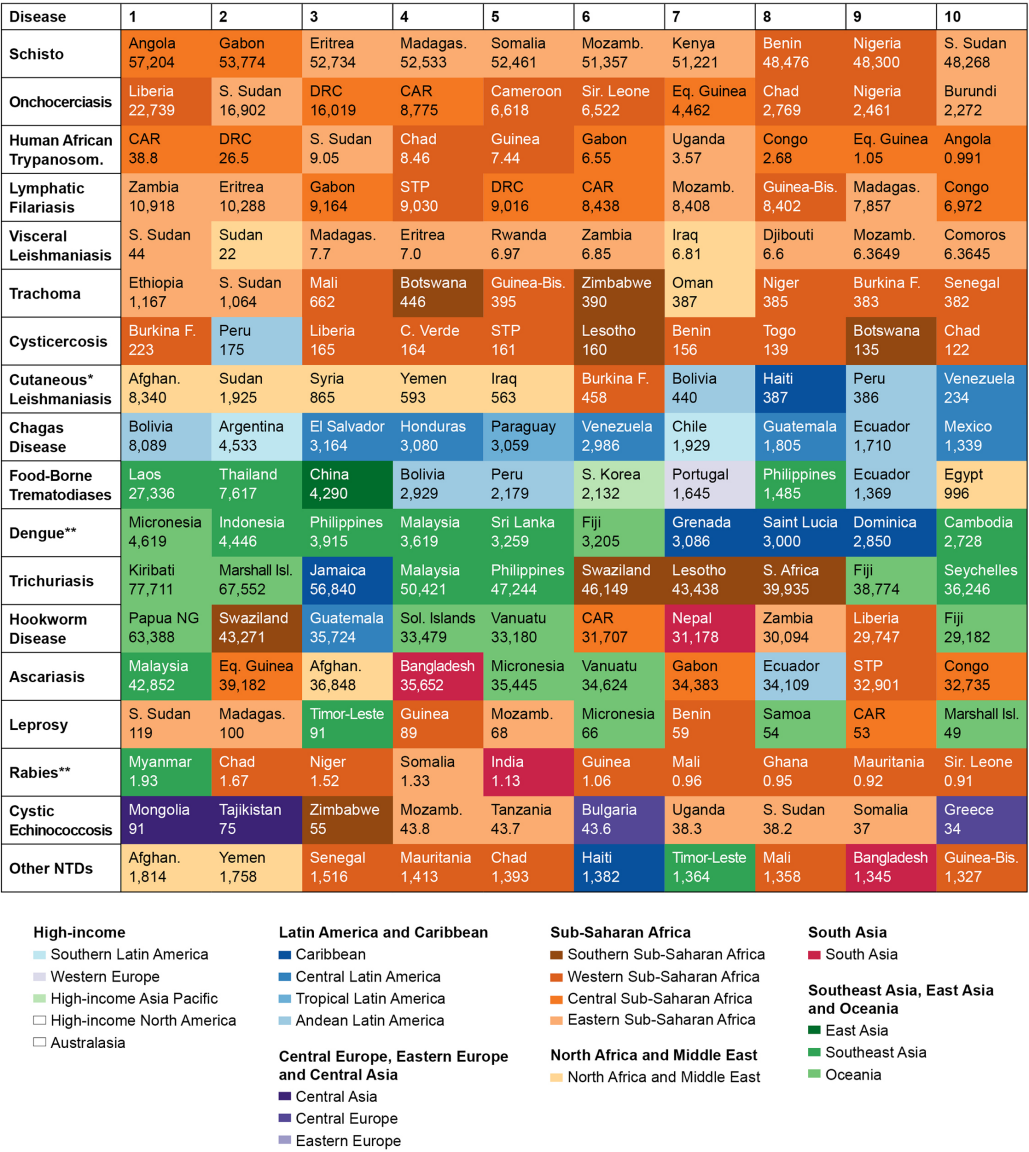
Countries with highest NTD prevalence in 2013. Countries with the highest prevalence (per 100,000 population) for the diseases indicated and the estimated prevalence in each country. Countries are color coded by Global Burden of Disease Study (GBD) regions. **Abbreviations:** CAR, Central African Republic; DRC, Democratic Republic of the Congo; STP, São Tomé and Principe. *Also includes mucocutaneous leishmaniasis, **Incidence rather than prevalence.

**Fig 4:**
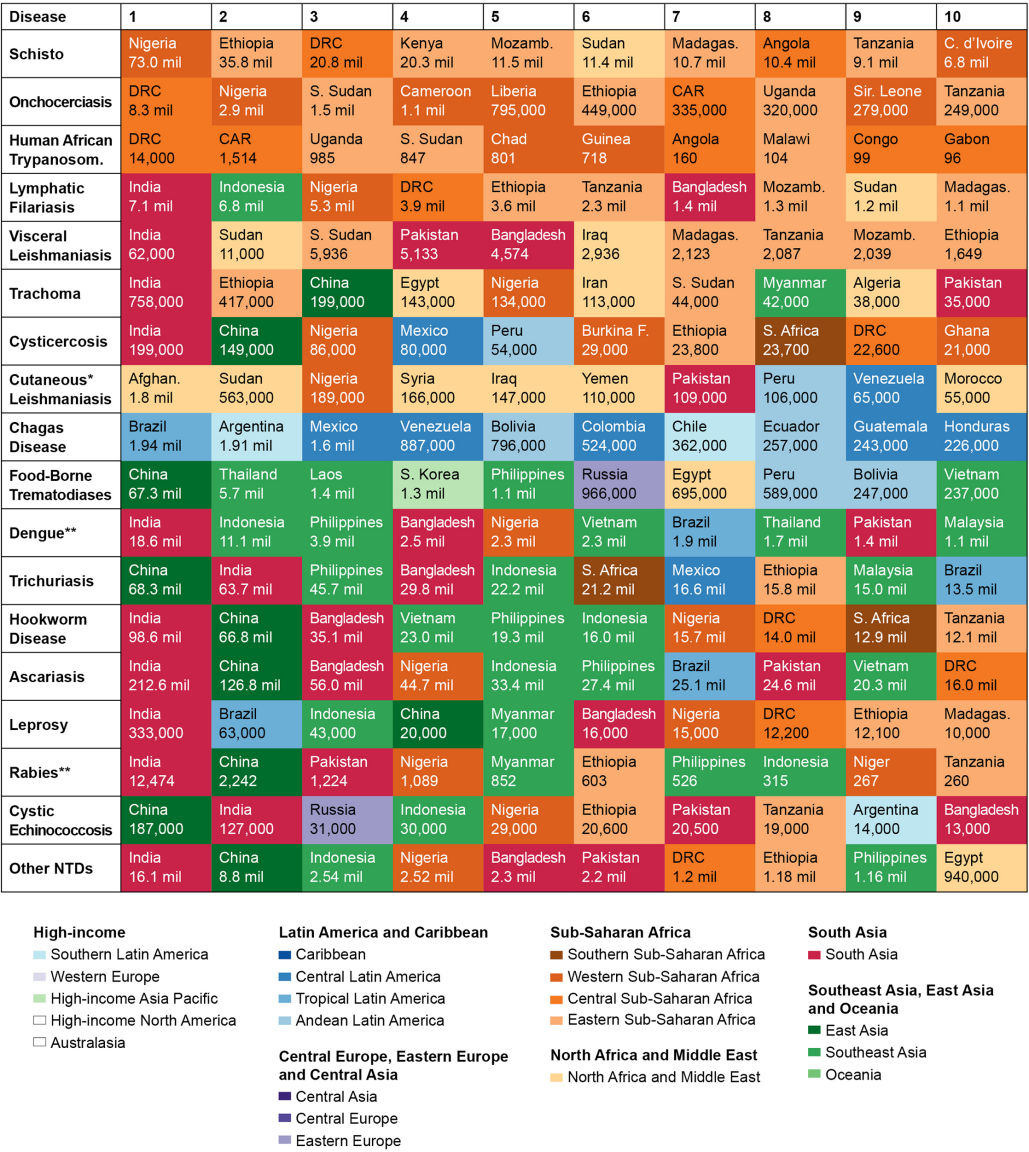
Countries with highest absolute number of NTD cases in 2013. Countries with the highest number of absolute cases for the diseases indicated and the estimated numbers in each country. These data are also available from the Institute for Health Metrics and Evaluation (IHME) website. Countries are color coded by Global Burden of Disease Study (GBD) regions. **Abbreviations:** CAR, Central African Republic; DRC, Democratic Republic of the Congo; STP, São Tomé and Principe. *Also includes mucocutaneous leishmaniasis, **Incident cases rather than prevalent cases, NOTE: As in Table 1, only symptomatic cases are estimated for dengue, trachoma, cystic echinococcosis, cysticercosis, and rabies.

GBD 2013 further identified the regions most affected by NTDs. Figs 3 and 4 highlight the disease-endemic countries burdened with either the highest prevalence (prevalent cases per 100,000 population), incidence (incident cases per 100,000 person-years) (Fig 3), or absolute number of cases (Fig 4) of NTDs. As one would expect, the burden of disease in DALYs was closely correlated to the number of cases.

As shown in Fig 4, India has the greatest number of cases of at least 10 different NTDs, followed by China (3), Democratic Republic of Congo (DRC) (2), and 1 each for Afghanistan, Brazil, and Nigeria. These numbers, while reflective of absolute burden, conflate disease prevalence and population size. By contrast, Fig 3 highlights areas where the prevalence of infection are highest, including less populous countries in sub-Saharan Africa, the Middle East and North Africa (MENA), Southeast and Central Asia, and the surprisingly high prevalence of helminth infections in Oceania. We next provide specific observations for each of the major NTD categories.

### Prevalence of helminth infections

The prevalence of the STH infections—trichuriasis, hookworm infection, and ascariasis—is especially high in Oceania, Southeast Asia, and South Asia. Large middle-income countries such as India and China as well as Bangladesh, Indonesia, and the Philippines stand out for having the largest numbers of cases of these infections.

While India has the largest number of LF cases (with infections estimated at 7.1 million), LF prevalence is highest in Zambia and Eritrea. China and the Southeast Asian countries of Thailand and Laos exhibit the largest number of cases of foodborne trematodiases (FBT), with 67.3 million cases in China alone. Thailand and Laos also have the greatest prevalence of FBT. The largest numbers of cases of schistosomiasis infection are in Nigeria with 73 million cases, followed by Ethiopia, the DRC, and Kenya, while countries with the highest prevalence of schistosomiasis infection are Angola and Gabon of central sub-Saharan Africa followed by several countries in eastern sub-Saharan Africa. The DRC leads in the number of cases of onchocerciasis infection, estimated at 8.3 million, and also has the third highest prevalence of infection behind Liberia and South Sudan. India and China lead the world in cysticercosis and cystic echinococcosis disease cases with over 100,000 each, while Burkina Faso has the highest prevalence of disease from cysticercosis and Mongolia has the highest prevalence of disease from cystic echinococcosis.

### Prevalence of protozoan infections

Among the 3 kinetoplastid infections, India has the largest number of visceral leishmaniasis (VL) cases at 62,000, although South Sudan and Sudan lead in prevalence. Brazil and Argentina have the largest number of Chagas disease infections with nearly 2 million cases each, while Bolivia has by far the highest prevalence—over 8,000 cases per 100,000 people. DRC has the largest number of absolute cases of prevalent and incident infections from HAT, estimated at 14,000 and 10,700, respectively. The Central African Republic had the highest prevalence and incidence of HAT infections in 2013.

### Incidence and prevalence of viral and bacterial infections

India leads the world in number of dengue fever cases with 18.6 million followed by Indonesia with 11.1 million, with Oceania and Southeast Asian countries leading in terms of incidence. India has the largest number of trachoma cases at about 758,000, followed by Ethiopia, with the Sahelian nations of Ethiopia, South Sudan, and Mali leading in terms of prevalence. It is important to note here that GBD 2013 estimates for this disease only represent the prevalence of blindness and vision impairment due to trachoma. The largest numbers of rabies cases occur in India (with over 12,000), China, Pakistan, and Nigeria, while Myanmar and the Sahelian nations of Chad, Niger, and Somalia lead in terms of incidence. India, Brazil, and Indonesia have the largest number of prevalent cases of leprosy in the world at 333,000, 63,000, and 43,000, respectively, as well as incident cases. South Sudan and Madagascar lead in terms of disease prevalence, while the Oceanic countries of the Marshall Islands, Federated States of Micronesia, and Kiribati lead in terms of disease incidence.

### Prevalence of other NTDs

Included in the group of “other NTDs” are a variety of diseases ranging from arthropod-borne viral infections to bacterial relapsing fevers to unspecified protozoan diseases and a variety of helminthic diseases for which limited disease burden data are available. Nevertheless, these diseases have an enormous impact. India has the highest number of cases of these diseases at 16.1 million, followed by China and Indonesia. Afghanistan and Yemen lead in terms of disease prevalence rates, followed by countries in western sub-Saharan Africa.

## NTD-associated deaths in 2013

Table 2 shows the estimated numbers of deaths and the age-standardized death rates in 2013 due to the 17 NTDs prioritized by WHO and other NTDs.

**Table 2:** Number of deaths and age-standardized death rates for NTDs in 2013 with percent change from 1990 to 2013 [4].

**NTDs**	**Deaths in 2013**	**Percent change since 1990**	**Age-standardized deaths per 100,000 in 2013**	**Percent change since 1990**
Visceral leishmaniasis	62,500	19.8%	0.86	−0.3%
Rabies	23,500	−38.3%	0.34	−54.0%
Chagas disease	10,600	−19.3%	0.17	−51.7%
Dengue	9,100	−1.3%	0.13	−13.6%
African trypanosomiasis	6,900	−69.7%	0.08	−78.9%
Schistosomiasis	5,500	−68.2%	0.08	−80.7%
Ascariasis	4,500	−50.7%	0.06	−54.7%
Cystic echinococcosis	2,200	−45.0%	0.03	−60.8%
Cysticercosis	700	−28.6%	0.01	−53.0%
Hookworm*	0	NA	0	NA
Trichuriasis*	0	NA	0	NA
Foodborne trematodiases*	0	NA	0	NA
Lymphatic filariasis*	0	NA	0	NA
Onchocerciasis*	0	NA	0	NA
Cutaneous/mucocutaneous leishmaniasis*	0	NA	0	NA
Trachoma*	0	NA	0	NA
Leprosy*	0	NA	0	NA
Other NTDs	16,300	−54.4%	0.24	−62.3%
**Total deaths from NTDs**	**141,800**	**NA**	**NA**	**NA**
**Additional neglected diseases**	**Deaths in 2013**	**Percent change since 1990**	**Age-standardized deaths per 100,000 in 2013**	**Percent change since 1990**
Typhoid fever	160,700	−10.8%	2.21	−25.9%
Venomous animal contact	57,200	−25.0%	0.82	−51.8%
Cholera	69,900	−44.3%	0.97	−51.9%
Paratyphoid fever	54,300	−14.9%	0.75	−28.0%
Cryptosporidiosis	41,900	−57.8%	0.58	−59.8%
Amoebiasis	11,300	−39.1%	0.18	−58.3%
Scabies*	0	NA	0	NA
Trichomoniasis*	0	NA	0	NA
**Total deaths from additionalneglected diseases**	**395,300**	**NA**	**NA**	**NA**

*Negligible evidence of deaths according to the Global Burden of Disease Study (GBD) 2013.

NOTE: For information on percent change calculations, see GBD 2013 capstone paper on mortality [4]. The estimates presented in this table are also available from the Institute for Health Metrics and Evaluation (IHME) website and previously published in [4]. **Abbreviations:** NA, non-applicable

In all, it was estimated that 141,800 deaths could be attributable to the 17 NTDs prioritized by the WHO plus “other NTDs” in 2013. However, if the additional NTDs such as typhoid fever, cholera, paratyphoid fever, cryptosporidiosis, and amoebiasis are also included among the diseases in Table 2, together they are estimated to have caused over 500,000 deaths in 2013, roughly equivalent to the number of deaths from all motor vehicle road injuries or breast cancer and more than half of the malaria deaths [4]. The leading NTD killers in 2013 were VL, rabies, and Chagas disease. Among those neglected diseases not prioritized by the WHO in 2013, typhoid fever, cholera, and venomous animal contact were responsible for the largest number of deaths. A particularly disturbing trend was noted for VL, for which the number of cases, deaths, and YLDs have increased since 1990 (Tables 1–3). Rates, however, have been effectively static, suggesting that the increase in absolute numbers may also be due to demographic changes such as population growth and changes in population age structure. However, this finding also shows how little progress has been made in fighting this infection.

**Table 3:** Leading causes of disability-adjusted life years (DALYs) resulting from the NTDs according to the Global Burden of Diseases Study (GBD) 2013 with attributing years lived with disability (YLDs) and years of life lost (YLLs) [2–4].

**NTD**	**DALYs (in millions) in 2013**	**Percent change for DALYs 2005–2013**	**YLDs (in millions) in 2013**	**YLLs (in millions) in 2013**
Visceral leishmaniasis	4.24	8.7%	0.008	4.23
Foodborne trematodiases	3.63	14.6%	3.63	0
Schistosomiasis	3.06	−13.9%	2.86	0.2
Hookworm	2.18	−0.5%	2.18	0
Lymphatic filariasis	2.02	−14.3%	2.02	0
Ascariasis	1.27	−29.0%	0.93	0.34
Rabies	1.24	−14.6%	0.0001	1.24
Onchocerciasis	1.18	−19.4%	1.18	0*
Dengue	1.14	17.0%	0.56	0.58
Trichuriasis	0.58	−12.3%	0.58	0
African trypanosomiasis	0.39	−54.3%	0.005	0.38
Chagas disease	0.34	4.6%	0.10	0.24
Cysticercosis	0.34	−16.4%	0.31	0.03
Cystic echinococcosis	0.18	−14.1%	0.08	0.1
Trachoma	0.17	−18.1%	0.17	0
Cutaneous and mucocutaneous leishmaniasis	0.04	35.9%	0.04	0
Leprosy	0.04	8.6%	0.04	0
Other NTDs	3.13	−11.8%	2.26	0.87
**Total NTDs**	**25.17**	**NA**	**16.95**	**8.21**
**Additional neglected diseases**	**DALYs (in millions) in 2013**	**Percent change for DALYs 2005–2013**	**YLDs (in millions) in 2013**	**YLLs (in millions) in 2013**
Typhoid fever	11.13	−13.7%	0.16	10.97
Cholera	5.17	−20.1	0.04	5.13
Paratyphoid fever	3.82	−8.0%	0.04	3.78
Cryptosporidiosis	3.46	−29.6	0.19	3.27
Venomous animal contact	3.00	−3.4%	0.15	2.85
Scabies	1.71	4.8%	1.71	0
Amoebiasis	0.38	−23.8%	0.04	0.34
Trichomoniasis	0.11	8.2%	0.11	0
**Total deaths from additional neglected diseases**	**28.78**	**NA**	**2.44**	**26.34**

NOTE: For information on percent change calculations, see the Global Burden of Disease Study (GBD) 2013 capstone paper on DALYs [2]. The estimates presented in this table are also available on the Institute for Health Metrics and Evaluation (IHME) website and were previously published in [2–4]. Information on DALYs and YLDs for Cholera, Cryptosporidiosis, and Amoebiasis is not available from IHME website or capstone papers. **Abbreviations:** NA, non-applicable

**Fig 5:**
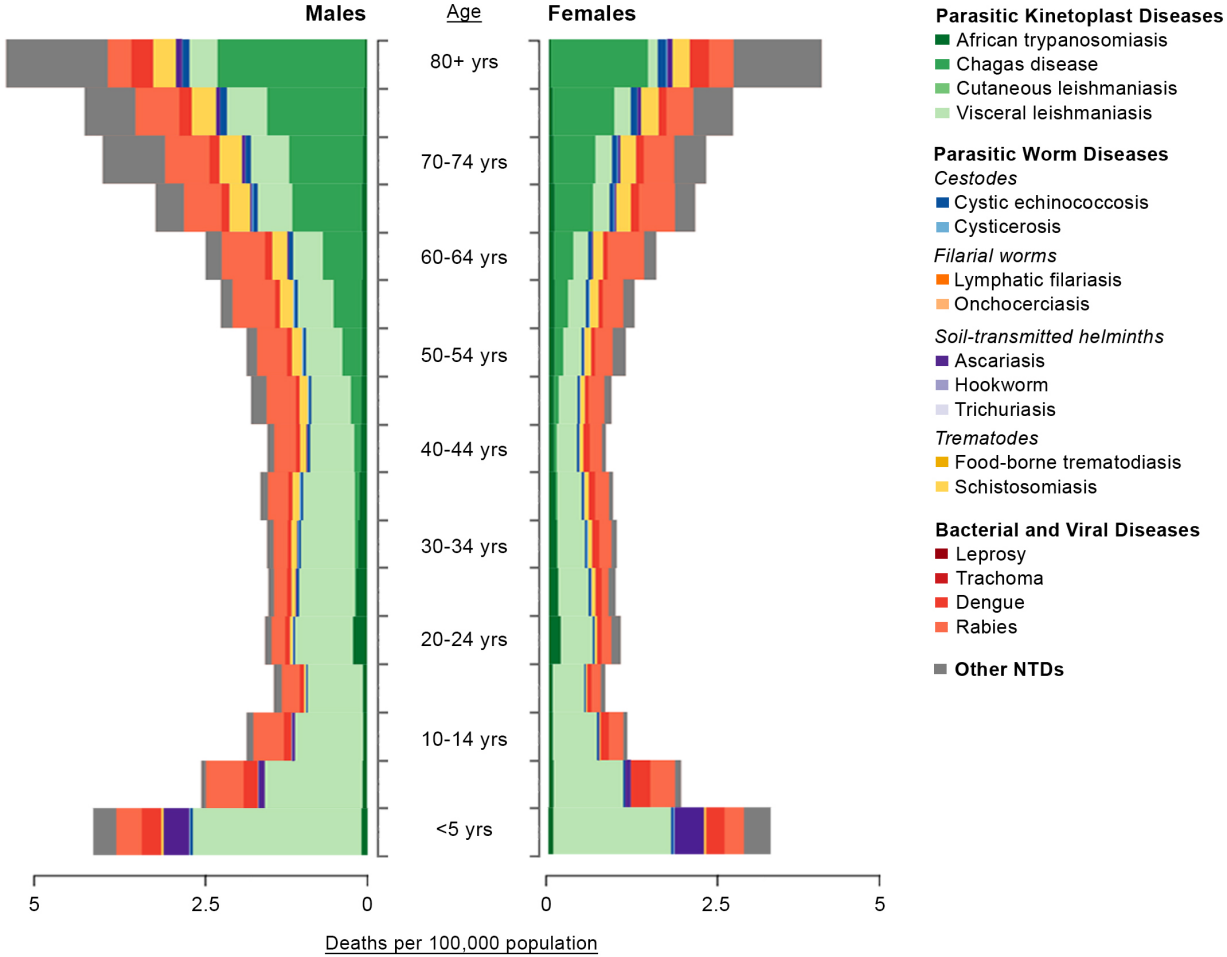
Deaths per 100,000 population by age and sex in 2013. The data used to generate this figure are also available on the Institute for Health Metrics and Evaluation (IHME) website.

When considered in terms of age and sex, the highest mortality caused by NTDs is also due to VL, primarily in the young, and Chagas disease, primarily in the elderly (Fig 5). “Other NTDs” also caused higher mortality with increased age. Rabies caused substantial mortality across all ages and ascariasis was primarily a cause of mortality for children under 5 years of age. In general, for the other NTDs listed, mortality is highest in the youngest and oldest populations.

## DALY trends for NTDs in 2013

As shown in Table 3, the GBD 2013 estimates for NTDs result in approximately 25 million DALYs, which is greater than the DALYs attributable to liver cancer, for instance [2]. The leading NTDs in terms of DALYs include VL, foodborne trematodiases, schistosomiasis, hookworm disease, and LF [2]. Among the additional neglected diseases, typhoid fever and cholera each cause more DALYs than VL. In addition, Table 3 shows the global burden of these diseases in terms of YLDs and YLLs. Onchocerciasis, the sixth highest cause of YLDs, was ranked highly in Liberia, Cameroon, and South Sudan in the top 10 leading causes of YLDs by country, predominantly due to onchocercal skin disease [3]. For most NTDs, YLDs account for a greater proportion of DALYs than do YLLs, and the most prevalent diseases (see Table 1) are also the ones that cause the most disability. In total, NTDs were responsible for 17 million YLDs and 8 million YLLs in 2013.

**Fig 6:**
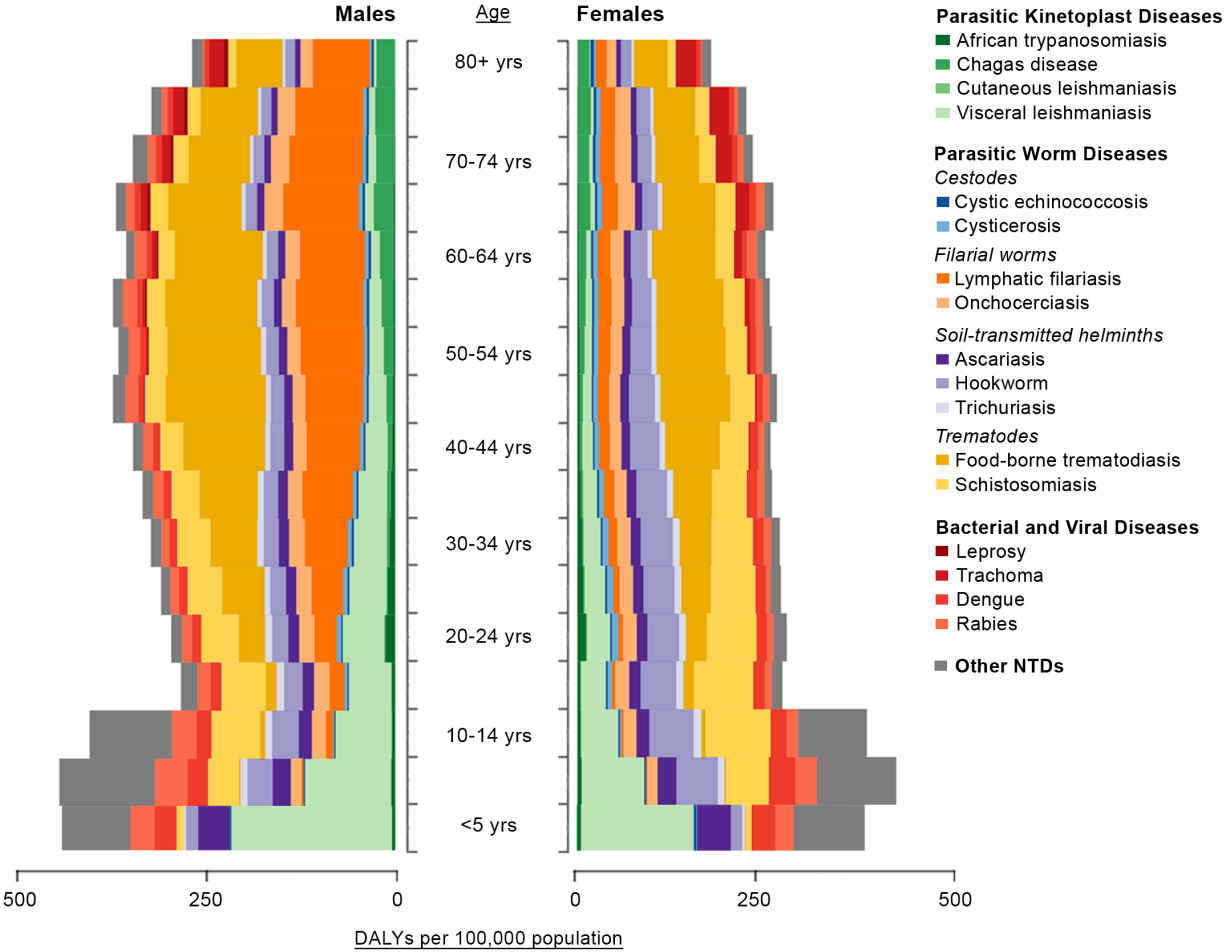
Disability-adjusted life years (DALYs) per 100,000 population by age and gender in 2013. The estimates used to generate this figure are also available on the Institute for Health Metrics and Evaluation (IHME) website.

Evaluating the etiologic composition of DALYs by age, we see that VL dominates among the very young (<5 years), but ascariasis, dengue, rabies, and “other NTDs” are also important NTDs among pediatric age groups (Fig 6). For older school-aged children and adolescents, STH infections and schistosomiasis are the leading causes of DALYs. Among adults, foodborne trematodiases, LF (especially in males), and hookworm infection represent some of the highest disease burdens due to NTDs. Among adolescent and adult women, schistosomiasis and hookworm infection are also leading causes of DALYs. For hookworm infection, it is likely that the adult-onset DALYs are linked to its high prevalence among adults and the associated high risk of anemia in pregnant and lactating women [16]. Further, DALYs for schistosomiasis may have been even higher if the GBD 2013 considered female genital schistosomiasis, perhaps Africa’s most common chronic gynecological disease, in these estimates [16].

**Fig 7:**
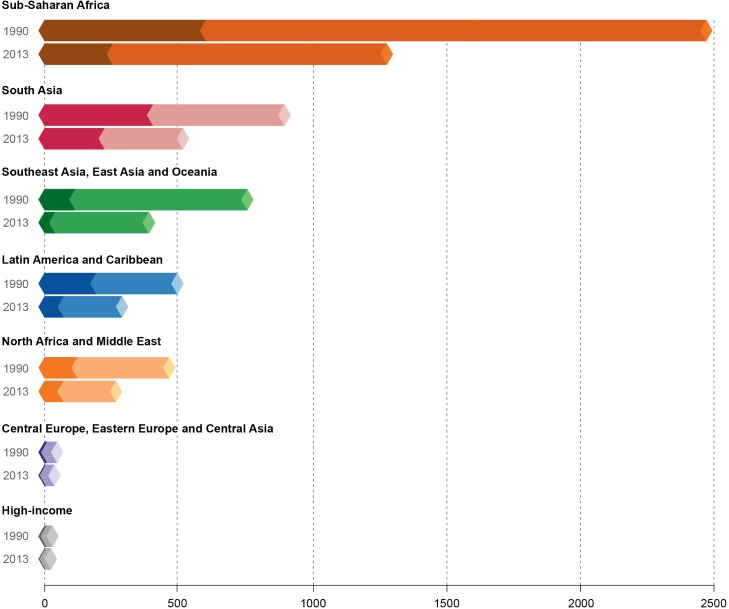
Proportional representation of disability-adjusted life years (DALYs) (per 100,000 population) from all NTDs combined in 1990 and 2013 by Global Burden of Disease Study (GBD) super region. Each bar represents the total disability-adjusted life years (DALYs) per 100,000 population for WHO-prioritized NTDs combined by GBD super region, broken down by percentage of years of life lost (YLLs) (darker shading) and years lived with disability (YLDs) (lighter shading). GBD-estimated numbers for DALY, YLL, and YLD rates for each super region in 1990 and 2013 are shown in the key.

Finally, Fig 7 shows the geographic distribution of DALYs per 100,000 population in 1990 and 2013 from all NTDs listed in Figs 3 and 4. Decreases were noted for each region from 1990 to 2013. The darker shading represents the portion of YLLs for these diseases, while the lighter shading represents the YLDs (by percentage of the DALYs). In each region, it is clear that the overwhelming majority of NTD-attributable DALYs arise from YLDs. Interestingly, the DALYs in South Asia are composed of almost equal numbers of YLDs and YLLs, likely due to the high prevalence of VL and other fatal NTDs in that region. In most super regions, especially those with the most DALYs from NTDs, DALYs were nearly halved from 1990 to 2013. While this clearly represents progress, Fig 7 also makes it clear that there is a lot of work to be done to reduce the substantial burden of these diseases, especially in sub-Saharan Africa and throughout Asia.

## Considerations and limitations of the GBD 2013 results for NTDs

Our overall objective in this article is not to provide an in-depth critique of GBD 2013 methodology or data but rather to highlight findings that we consider of importance for the NTDs community. The GBD category of “other NTDs” includes a range of other neglected tropical diseases (relapsing fevers, typhus fever, spotted fever, Q fever, other rickettsioses, other mosquito-borne viral fevers, unspecified arthropod-borne viral fever, arenaviral haemorrhagic fever, toxoplasmosis, unspecified protozoal disease, taeniasis, diphyllobothriasis and sparganosis, other cestode infections, dracunculiasis, trichinellosis, strongyloidiasis, enterobiasis, and other helminthiases) but these are not modeled separately. No information from the GBD 2013 is currently available for Buruli ulcer, chikungunya virus (included under “other NTDs”), and yaws. Unless stated otherwise, estimates presented are for both symptomatic and asymptomatic cases.

Countries with the lowest NTD burden often lie within the high-income super region. However, we have noted previously that surprisingly high rates of NTDs also occur among the poorest residents of the world’s largest economies: the Group of 20 nations (G20) plus Nigeria and other wealthy countries in the MENA, Asia, and the Americas (the concept of “blue marble health”) [17]. The GBD 2013 confirms that high NTD burden occurs within the G20 nations and Nigeria [18]. However, gaps in the estimates remain. For example, GBD 2013 estimates for Chagas disease were restricted to endemic countries, and no estimates were made of imported cases in countries with large Latin American immigrant populations, such as the United States and Spain. However, the U.S. Centers for Disease Control and Prevention (CDC) estimates that there are at least 200,000 cases of Chagas disease in the US, which would place the US as the country with among the highest number of cases in the world [19]. In addition, there is evidence of triatomine insects infected with *Trypanosoma cruzi* and positive for human blood, as well as autochthonous transmission of Chagas disease in the US, especially in Texas [20]. Unfortunately, reporting of Chagas disease in the US is low, likely due to a lack of healthcare provider knowledge of the disease [21]. The exclusion of imported cases from nonendemic countries as well as underreporting of autochthonous transmission suggests that GBD 2013 is underestimating Chagas prevalence globally.

Also of interest is the impact of MDA for intestinal helminth infections, schistosomiasis, LF, onchocerciasis, and trachoma, which has been integrated and expanded on a global scale beginning in 2006 through financial support of the governments of the US (United States Agency for International Development’s NTD Program) and the United Kingdom [22]. Control through MDA started at different time points for different diseases and countries, so progress has been heterogeneous [23]. For example, large-scale vector control for onchocerciasis (Onchocerciasis Control Programme in West Africa, OCP) started in West Africa in the mid-1970s. From 1995 onwards, the African Programme for Onchocerciasis Control (APOC) coordinated the gradual scale-up of MDA with ivermectin in the remaining endemic African countries and the OCP used ivermectin to control any recrudescence. By now, a majority of areas in need of treatment for onchocerciasis are receiving MDA [24–26]. The Global Program to Eliminate Lymphatic Filariasis (GPELF) has been in place since 2000, while large-scale treatment for STH infections and schistosomiasis started later [27]. Following its successful large-scale use in Morocco, MDA of azithromycin has been included as part of WHO’s surgery, antibiotics, facial cleanliness, and environmental improvement (SAFE) strategy for eliminating trachoma [28–32]. The trends we see for the major helminth infections may be potentially explained by the relatively recent start of widespread schistosomiasis control programs and low single-dose drug efficacies for hookworm and trichuriasis [33, 34], given that we are already seeing substantial reductions in ascariasis, as highlighted above.

We also point out that prevalence estimates for HAT and leprosy for the GBD 2013 are derived in part from reported incidence figures and literature-based assumptions about the natural history of these diseases.

It is important to note that the GBD 2013 cysticercosis estimates only include cases of neurocysticercosis (NCC)-associated epilepsy, although this brain infection may cause several other neurological disorders [35]. The GBD estimates are based on the estimated prevalence of secondary epilepsy and the prevalence of NCC among people living with secondary epilepsy. Because not all infections of cysticercosis result in NCC, it is likely that there are more cysticercosis cases than those estimated by GBD 2013. Likewise, not all cases of NCC are associated with epilepsy but rather with severe chronic headaches, stroke, focal deficit, and dementia, to name a few conditions. Because such cases were not included in the current estimates, this may have led to an underestimation of the burden of cysticercosis by the GBD 2013. Moreover, this means that part of the disability incurred by other neurological and mental health disorders caused by NCC increases the DALYs of these diseases, making other disorders look less important. Cysticercosis estimates are based on sparse literature data on NCC prevalence among people with epilepsy (from 12 countries: Bolivia, Brazil, Burkina Faso, Colombia, Ecuador, Guatemala, Honduras, India, Mexico, Peru, South Africa, and Tanzania) combined with country-level covariate data on access to sanitation and proportion of the population that is Muslim. It has been pointed out appropriately by 1 of the reviewers for this manuscript that intermediate-host pig populations should be considered rather than a proportion of Muslim populations in order to adequately capture nations such as Chad, Ethiopia, and Sudan that have significant non-Muslim populations yet also have very few pigs. Unfortunately, none of these indicators measure precisely the risk factor of most interest for NCC, which is the exposure of humans to livable *Taenia solium* eggs in the environment. Such exposure, in turn, depends on the level of sanitation and the prevalence of human (*T*. *solium*) taeniasis in the population. The prevalence of taeniasis depends in turn on the consumption of undercooked pork meat. Therefore, although the presence of pigs may act as an indirect yet important indicator of NCC, it is not the only one. Other factors also play a role (people might eat well-cooked pork, which is common when the meat is consumed at home, or pigs may not ever be exposed to human feces in areas where their access to human feces is restricted). Better estimates of the burden of NCC will be feasible as more data on the actual prevalence of NCC-associated neurological disorders becomes available with the development of better diagnosis for infections of the brain [36]. Another challenge is that the very clustered nature of cysticercosis, NCC, and taeniasis makes it difficult to generalize data from small-scale studies to larger areas, making it difficult to evaluate the true burden of cysticercosis.

Similar to cysticercosis, the GBD 2013 cystic echinococcosis estimates relied heavily on modeling approaches to fill in data gaps. While this method does allow for a regional picture of where the condition is more prevalent, many individual country-level estimates will require additional verification and refinement. One example of where country-level estimates will need to be improved is in Asia. While portions of China and Central Asia are known to be highly endemic for cystic echinococcosis, most countries in Southeast Asia (e.g., Indonesia, Thailand, and Vietnam) are believed to be nonendemic for this disease. This observation is evidenced by both the lack of reports of autochthonous human cases from these countries and no reported animal infections. However, based on GBD country-level covariate information used to fill in data gaps, the numbers of estimated cases in these countries appear to be high, whereas current data indicate that there are few or no cases. The surprisingly high cystic echinococcosis case numbers predicted for Indonesia are an example of this phenomenon.

In 2010, the WHO Foodborne Disease Burden Epidemiology Reference Group (FERG) released their own calculations for the burden of foodborne diseases such as cysticercosis and cystic echinococcosis [37]. The difference in DALY estimates for some diseases between the FERG estimates and GBD 2010 were striking [2, 38]. For cysticercosis, the GBD 2010 estimated 514,000 DALYs, whereas the FERG study estimated over 2.7 million DALYs. The GBD 2013 estimates 340,000 DALYs for cysticercosis—still far off from the FERG estimates. The FERG study used some of the same input data as GBD study, similar analytical methods, and many of the same weightings [39]. However, some different and important choices were made. In the case of cysticercosis, the FERG study allocated a much larger proportion of the epilepsy burden to cysticercosis, based on data from a systematic review [40]. Such differences may be considered important with respect to assessing changing burden over time and highlight the need to align methodologies in an open and transparent fashion.

For several NTDs, the number of deaths reported by the GBD 2013 is also likely to represent an underestimate. For example, urogenital schistosomiasis is a major cause of renal disease and bladder cancer in Africa and the Middle East [41], and yet only 5,500 deaths were ascribed to all of the world’s 290 million schistosomiasis cases. It is likely that many of the schistosomiasis-related deaths are being classified in categories such as chronic kidney disease or bladder cancer, now linked to 956,200 deaths and 173,900 deaths, respectively [4]. Also, despite the official classification of *Opisthorchis viverrini* and *Clonorchis sinensis* as Group 1 carcinogens causing highly fatal cholangiocarcinoma [42], there were no estimated cases of deaths attributed to foodborne trematodiases. Previous estimates resulted in 7,000–8,000 deaths annually due to cholangiocarcinoma caused by these 2 foodborne liver fluke species, and even these numbers were considered too low [38, 43, 44]. However, it is likely again that these deaths were classified as cancer deaths and not as deaths due to foodborne trematodiases. Similarly, there are 183,400 deaths ascribed to iron-deficiency anemia [4], of which hookworm disease is a major cause [45], and yet no deaths are attributed to this NTD in the GBD 2013. These and other factors may result in underreporting of deaths due to diseases such as schistosomiasis and hookworm infection. Estimates of dengue may also be too low. The GBD 2013 estimates for deaths from dengue virus range from 8,365 in 1995 to 10,394 in 2010, but in 2013, they decreased to 9,100. Considering the dramatic increase in dengue incidence and geographic spread of dengue transmission seen in recent years as well as recent evidence on the underreporting of dengue deaths in well-funded surveillance systems, the number of deaths due to dengue virus may be substantially higher than that estimated by the GBD study for the year 2013 [46–49]. A recent systematic review and meta-analysis suggests that estimates of mortality attributed to Chagas disease may also be low [50]. Similarly, the GBD 2013 estimates 23,500 rabies deaths, but another recent estimate also based on modeling and extensive literature review estimated 59,000 annual deaths from rabies [51].

For similar reasons, the GBD 2013 also likely underestimates the DALYs attributed to the NTDs, especially for schistosomiasis and hookworm disease [1]. The DALYs for scabies may also be an underestimate, as the indirect effects of streptococcal infection on renal and cardiovascular function may not be appreciated. Conversely, the DALY estimates for foodborne trematodiasis nearly doubled between GBD 2010 and GBD 2013, from 1.9 million to 3.6 million DALYs [1, 2]. This surge was caused in part by a revised disability weight for paragonimiasis. Efforts are underway to harmonize these changes in the upcoming GBD 2015. The corrected burden estimates for foodborne trematodiases might be in the 2.0–2.5 million DALYs range, which would also be in line with recently published WHO estimates [38]. For rabies, the GBD 2013 estimated 1.24 million DALYs, but similar to the death estimates, a recent review has estimated 3.7 million DALYs from rabies [51].

While the GBD 2013 provides timely and relevant NTD burden data, critical caveats need to be clearly stated and considered in the interpretation and application of these estimates. Among the most frequently mentioned and also critical gaps is the lack of highest-quality epidemiological data, which is not only an NTD-specific issue but of particular importance for this disease cluster. Another critical issue—again, not exclusive to NTDs—is the correct modeling of pathways from infection to disease and death and a correct attribution of the resulting YLDs, YLLs, and DALYs. Both aforementioned issues ask first and foremost for more primary data from NTD-specific research in order to strengthen the evidence base and the case of NTDs. However, there are also methodological decisions in the global burden of disease estimation which need to be carefully considered. For the sake of shortness, we would like to highlight just 2 of these methodological points which cover the spectrum from (1) practical issues that the NTD community can address immediately to improve the next generation of GBD estimates to (2) very fundamental decisions in the design of the DALYs, which the NTD community cannot directly influence within the massive GBD collaboration but can at least carefully observe and comment.

First, for many country estimates, the GBD disease modeling approach borrows strength from relevant data in neighboring/similar countries and from additional covariates from a massive covariate database. However, this may lead to some estimation errors, which are negligible at the global level but relevant at national scale. For instance, Australia is considered rabies free [52], but GBD reported 2 rabies deaths in Australia. GBD also reports 5 rabies deaths in the UK just in 2013. However, there have only been 4 rabies deaths in the UK since 2000 (all in individuals bitten by dogs when abroad). Identifying such inconsistencies and providing guidance to the GBD data analysis team (e.g., force certain country estimates to be strictly zero) would further improve the precision of GBD estimates. For example, as a potential strategy for other diseases, autochthonous human case reports on foodborne trematodiases have been reviewed and mapped by Fürst et al. Thereby, countries were classified (1) as having suitable national data that should be directly applied in burden estimation, (2) as having no suitable national data but case reports and where, consequently, national estimates should be predicted based on the data from similar countries and relevant covariates as the best option, and (3) as having no suitable national data, no case reports, and as being also not known for its endemicity, where the models should therefore not predict any cases [53].

Second, since GBD 2010, the GBD studies switched from incidence- to prevalence-based DALYs [54]. The exact effect of this fundamental decision on the burden estimation and the comparison of acute with chronic sequelae in populations experiencing varying dynamics is unclear. This is true in general and hence also for the NTD burden estimates. However, at least for some NTDs, the readers can refer to the WHO FERG estimates, which provide incidence-based DALY estimates, in order to obtain a more complete picture on the respective burden estimates [37–39].

Finally, terms such as prevalence or cases are not always clearly defined in GBD models for individual diseases, which can create some confusion when interpreting the meaning of the results. Future iterations of the GBD study will likely be more transparent, making interpretation simpler and comparison between estimates from other sources more clear.

## Concluding remarks

The GBD 2013 highlights reductions in the global prevalence of some specific NTDs such as LF, onchocerciasis, trachoma, and ascariasis, likely due to MDA, water, sanitation, and hygiene (WASH) and other control measures, and HAT reductions, likely due to expanded efforts for case detection and treatment elimination strategies, especially for the Gambian form of the disease [10, 15]. In contrast, we have not seen meaningful declines in diseases such as hookworm infection, trichuriasis, and schistosomiasis, while for dengue, leishmaniasis, and foodborne trematodiases, we have seen substantial increases [3]. Therefore, we need to consider adopting public health policies to address these trends and adapt our current approaches to specifically guide better disease surveillance, improved water quality and sanitation, affordable diagnostic tests, access to healthcare and medications, and further investments in new preventive and disease-control technologies. We also need to look at shaping NTD control policies in the countries where NTDs are highest, which include large middle-income countries such as India, China, and Brazil, where income inequality and the resulting inequality in access to healthcare, safe housing, clean water, and sanitation has allowed these diseases to persist despite economic growth [17]. However, GBD 2013 also highlights the high prevalence of NTDs in some of the smaller conflict-ridden nations and nations in a postconflict period. For example, Liberia, Central African Republic, South Sudan, and Afghanistan lead the world in several NTD categories. Creating new strategies to fight NTDs in such countries, which often have highly fractured health infrastructure and struggle to keep hospitals open, poses a different, perhaps more difficult policy challenge, but is one that should not be ignored. High NTD prevalence was also noted in several Oceanic and Southeast Asian countries and should also be addressed.

While there are some concerning trends revealed by the GBD 2013, we should not overlook or downplay the major achievements so far. During the 23 years from 1990 to 2013, much progress has been made in reducing the prevalence and burden of several NTDs. In the year 2000, the United Nations Millennium Development Goals (MDGs) spurred action against human immunodeficiency virus, tuberculosis, and malaria. Those actions have paid off in ensuing years as we are making continued progress in fighting “the big three.” However, goals specifically targeting NTDs were notably missing from the MDGs beyond a mention of the “other diseases.” This omission sparked a response from a small group of dedicated NTD activists to raise the profile of NTDs in the global health community. Since then, we have seen the creation of a new Department of NTDs at WHO, a Global Network for NTDs, the creation of NTD research and support centers, and the establishment of programs to support MDA at the United States Agency for International Development and the British Department for International Development [22]. In addition, several product development partnerships (PDPs) have formed to develop new NTD drugs, diagnostics, and vaccines, and moreover, major pharmaceutical investment in a dengue vaccine has resulted in the first dengue vaccine currently approved in 3 countries as of January 2016. An open access scientific journal dedicated solely to NTDs began publication in 2007. The first WHO report on NTDs in 2010 was followed by a roadmap action plan in 2012, the launch of the END Fund and the London Declaration the same year, and a specific resolution for NTDs from the World Health Assembly in 2013. Such efforts have continued beyond 2013 with agreements such as the Addis Ababa NTD Commitment signed at the end of 2014, establishment of the NTD Modeling Consortium, and the recent inclusion of NTDs in the new UN Sustainable Development Goals. As a result of these joint efforts, some countries have successfully eliminated certain endemic NTDs. For example, Mexico was declared to have eliminated onchocerciasis in 2015 following its elimination in Colombia and Ecuador in 2013 and 2014, respectively [12].

Overall, the results presented here indicate that, despite significant gains, much work remains in the fight against NTDs. There are still approximately 2.3 billion cases of NTDs, which cause a substantial global disease burden. It is critical that we as a global community continue our efforts to help end the suffering caused by NTDs. Helping nations to achieve health for the poorest of their citizens will be a step forward in achieving their Sustainable Development Goals. Finally, most of the NTDs are still underreported, and the quantification of their burden is limited by the data that are available. Therefore, screening and notification efforts for the NTDs should be increased in order to capture the true burden of these diseases. Understanding the true burden of NTDs is essential to track health progress, assess the impact of public health interventions, and inform evidence-based policy decisions.
